# Understanding the Role of Mono and Ternary Alkali Metal Salts on CO_2_ Uptake of MgO Sorbents

**DOI:** 10.3390/ma16247539

**Published:** 2023-12-06

**Authors:** Patrícia Correia, Carla I. C. Pinheiro, Paula Teixeira

**Affiliations:** Centro de Química Estrutural, Institute of Molecular Sciences, Departamento de Engenharia Química, Instituto Superior Técnico, Universidade de Lisboa, Av. Rovisco Pais 1, 1049-001 Lisboa, Portugal; ana.patricia.correia@tecnico.ulisboa.pt (P.C.); carla.pinheiro@tecnico.ulisboa.pt (C.I.C.P.)

**Keywords:** MgO-based sorbents, alkali metal salts, intermediate-temperature carbon capture

## Abstract

CO_2_ uptake by MgO-based sorbents at intermediate temperatures is attractive for pre- and post-combustion CO_2_ capture applications. However, besides the high CO_2_ uptake potential of these materials (1.1 g CO_2_ g^−1^ sorbent), in practice, the realistic CO_2_ capture is far from that of the theorical values. In this work, the sol–gel method was used to synthetize unsupported and supported MgO sorbents (10% Ca^−^ or 10% Ce^−^ support, mol) that were impregnated with different fractions (15, 25, and 35; % mol) of a NaNO_3_ single salt or a ternary alkali salt (NaNO_3_, LiNO_3_ and KNO_3_ (18/30/52; % mol)). To understand the role of alkali metal salts (AMSs) in the MgO sorbents’ performance, the working and decomposition temperature ranges of AMS under different atmospheres (CO_2_ and air) were evaluated. The findings show that the CO_2_ uptake temperature range and maximum uptake (20–500 °C, CO_2_ atmosphere) of sorbents are correlated. The cyclic CO_2_ uptake of the most promising sorbents was tested along five carbonation–calcination cycles. For the first and fifth cycles, respectively, the 15 (Na, K, Li)-MgO sorbents showed the highest carrying capacity, i.e., 460–330 mg CO_2_ g^−1^ sorbent, while for the 15 (Na, K, Li)-MgO-Ca sorbents, it was 375–275 mg CO_2_ g^−1^. However, after the first cycle, the carbonation occurred faster for the 15 (Na, K, Li)-MgO-Ca sorbents, meaning that it can be a path to overpassing carbonation kinetics limitations of the MgO sorbent, making it viable for industrial applications.

## 1. Introduction

MgO is an intermediate-temperature CO_2_ sorbent (200–400 °C) that gained relevance in the scientific community during the last decade [1,2]. This sorbent is suitable for both post- and pre-combustion CO_2_ capture technologies. Most of the studies in the literature have been performed for post-combustion conditions [3,4], but the application in sorption-enhanced water gas shift (SEWGS) reactions [5,6], i.e., under pre-combustion conditions, is also relevant since it increases the H_2_ production and purity.

CO_2_ capture is based on the following reversible chemical reaction: MgO (s) + CO_2_ (g) ⇄ MgCO_3_ (s), and since one mole of MgO can absorb one mole of CO_2_, its theoretical CO_2_ capture capacity is high (1.1 g CO_2_ g^−1^ MgO) [7]. The calcination and carbonation reactions are both dependent on temperature, and its equilibrium is also related to the CO_2_ partial pressure according to Equation (1) [8]:

(1) $$T_{eq}=\frac{13636}{ln\left(\frac{\left[bar\right]}{P_{{CO}_{2}}}\right)+20.01}-273.15$$

Calcination is an endothermic reaction (∆H 25 °C = +116.9 kJ mol^−1^) that, ideally, should be performed in an atmosphere rich in CO_2_ (>450 °C) to allow for a high CO_2_ concentration at the exit of the calciner with an efficient compression and storage for the utilization of the captured CO_2_. Some advantages of this sorbent, which is available in nature as MgCO_3_ or MgO, include its characteristics of being nontoxic and noncorrosive and its availability at a relatively low cost. Moreover, as mentioned above, its regeneration can occur at around 450–500 °C; then, the energy consumption during this step is reduced in comparison with that of CaO or Li_4_SiO_4_ sorbents’ regeneration that needs temperatures higher than 700 °C [5].

In addition to the interesting advantages of MgO as a CO_2_ sorbent, MgO has a very low experimental CO_2_ uptake capacity of 11–20 mg CO_2_ g^−1^ and very poor experimental sorption kinetics at a very favorable temperature of 200 °C from a thermodynamic point of view [9]. Some explanations have been proposed to justify the low performance of MgO [7]: (i) the low surface area does not allow for sufficient exposure of its basic sites for CO_2_ carbonation; (ii) the low porosity can obstruct the CO_2_ diffusion through the pores and delay the carbonation equilibrium; (iii) the MgO has a volume expansion of 2.49 due to the formation of MgCO_3_, while the formation of dense layers adjacent to basic active sites of the MgO sorbent can inhibit the CO_2_ carbonation; (iv) the MgO has an intrinsically high lattice enthalpy.

For an upscale application of MgO sorbents, the kinetic and carrying capacity limitations need to be overcome. In this sense, different paths to enhance the MgO sorbents performance, based on their dependence on intrinsic and extrinsic factors, have been assessed. To upgrade the internal properties of sorbents, the effects of distinct factors and their synergies have been evaluated: different MgO precursors, the synthesis of mesoporous MgO, dispersion on inert supports, and doping with alkali metal salts (AMSs). Factors like the CO_2_ concentration, pressure, presence of water, and presence of gas impurities are extrinsic factors that can also influence CO_2_ uptake by sorbents.

The most common magnesium precursors include inorganic salts, organometallic salts, and natural minerals. In general, the organometallic salts of magnesium are better precursors than the other two types in producing efficient MgO sorbents. This is mainly because the former usually have a larger molecular weight, which results in MgO with a better pore size and porous structure [2]. However, the organometallic precursors are expensive. On the contrary, natural Mg-containing minerals are abundant in nature and, thus, are cost-effective. For instance, magnesium oxalate (MgC_2_O_4_) is a better precursor than MgCO_3_ because it generates MgO sorbents with better performance [3]. Guo et al. [3] obtained a MgO sorbent derived from magnesium oxalate dihydrate (MgC_2_O_4_ · 2H_2_O) with the highest CO_2_ capture capacity of 194 mg CO_2_ g^−1^ sorbent. This sorbent is characterized by excellent textural properties, a uniform surface, and abundant basic sites. Hanif et al. [10] synthesized mesoporous MgO with a BET surface area higher than 350 m^2^ g^−1^ using the ammonium-hydroxide-assisted precipitation of Mg(OH)_2_ from a Mg(NO_3_)_2_ aqueous solution followed by the thermal degradation of the precipitated Mg(OH)_2_ in vacuum. This sample presented a CO_2_ capture capacity of 75 mg CO_2_ g^−1^ sorbent at 300 °C. The synthesis of mesoporous MgO is also an effective way to improve sorbents’ CO_2_ capture capacity. When the CO_2_ reacts with MgO, it forms a covering layer of the thermodynamically stable MgCO_3_ on the unreacted sorbents’ surface, which delays the CO_2_ molecules’ diffusion through the product layer. Thus, the production of an MgO sorbent with a high surface area and porosity should facilitate the CO_2_ diffusion, enhancing the capture capacity and kinetics reactivity of the sorbent [2]. The dispersion of MgO on a porous support also increases the surface area availability of well-exposed basic active sites, which in turn improves the sorbent’s capture capacity. Ideally, a good support must present a stable porous structure, which should enhance the sorbent performance and reduce its sintering after several carbonation–calcination cycles, contributing to the stability of the sorbent’s CO_2_ uptake capacity.

Among the approaches to improve the CO_2_ uptake properties of MgO, the doping with alkali metal salts is the most widely recognized promising approach. The aim of the most recent experimental works is to improve the CO_2_ uptake capacity of these materials up to 0.7–0.8 g CO_2_ g^−1^ sorbent [11]. The main categories of alkali doping described in the literature [2] are the alkali carbonate doping, alkali nitrate/nitrite doping and binary or ternary alkali doping.

A US patent [12] described that alkali metal carbonate-doped MgO sorbents prepared by coprecipitation registered CO_2_ capture capacities of 48–570 mg CO_2_ g^−1^ sorbent at the temperature range of 350 to 400 °C and under 70% dry CO_2_. Moreover, it was observed that the performance of the doped sorbent was influenced by the doping precursor. For instance, the MgO sorbents doped with Na_2_CO_3_ showed better performance than when doped with Li_2_CO_3_ or K_2_CO_3_.

Zhang et al. [13] doped MgO sorbents with NaNO_3_. The authors achieve a good CO_2_ carbonation kinetics and a MgO conversion of 75% against only 2% for an undoped MgO, both at 330 °C and ambient pressure. It was stated that molten NaNO_3_ decreases the dissociation energy of Mg-O ionic bonds in bulk MgO; thus, the molten NaNO_3_ acts as a phase transfer catalyst (PTC) between bulk MgO and CO_2_ molecules which, in turn, facilitates the carbonation reaction. In addition, molten alkali metal nitrates prevent the formation of a rigid, CO_2_ impermeable, and monodentate carbonate layer on the surface of MgO as it occurs with bare MgO and promote the rapid generation of carbonate ions to allow a high rate of CO_2_ uptake [2].

Concerning to binary or ternary alkali doping, very promising results are found in the literature. Lee et al. [14] studied the Na_2_CO_3_/NaNO_3_-doped MgO sorbent that maintained a CO_2_ sorption capacity of 153 mg CO_2_ g^−1^ sorbent after seven cycles. The authors related this performance with the interaction established between MgO, CO_2_ and the molten NaNO_3_ that results in the formation of the double salt Na_2_Mg(CO_3_)_2_. Identical results were registered for other cases of binary doping, such as K_2_CO_3_/KNO_3_-doped MgO sorbents. Zhang et al. [15] provided a detailed explanation for the enhancement of the CO_2_ uptake capacity of MgO sorbents doped with the binary Na_2_CO_3_/NaNO_3_ system. The mechanism is stated to be similar to that of the single alkali nitrate. The bulk MgO dissolves in the molten salt because Mg-O ionic bonds are easy to break. Na_2_CO_3_ also dissolves in the same liquid medium because carbonate salts have good solubility in molten salts. Hence, ion pairs of Mg^2+^, O^2−^ and CO_3_^2−^ are formed and react with the CO_2_ molecules, generating the double salt (Na_2_Mg(CO_3_)_2_). The same mechanism is appropriate to binary alkali nitrate/carbonate doping, which forms the CaMg(CO_3_)_2_ double salt [2].

The binary doping with alkali nitrate/nitrite is also an interesting matter of study. Zhao et al. [16] compared the CO_2_ sorption capacities of the single NaNO_3_ and of the binary NaNO_3_/NaNO_2_-doped MgO sorbents. The latter showed higher CO_2_ sorption capacity than the former. This new evidence was explained by the reduction in the melting temperature of the eutectic mixture. While single NaNO_3_ and NaNO_2_ present a theoretical melting point of 308 °C and 271 °C, respectively, the eutectic mixture of NaNO_3_/NaNO_2_ exhibits a melting temperature of 185 °C. Thus, the eutectic mixture facilitates the carbonation process by providing a molten phase that works like a liquid channel. Ternary doping with NaNO_3_, LiNO_3_ and KNO_3_ (18/30/52; % mol) registered a sharper reduction in the eutectic mixture’s melting point (120 °C) and an enhanced CO_2_ sorption performance [2].

Summarizing, the doping with AMS improves the cyclic CO_2_ sorption capacity of MgO sorbents when compared to that of commercial MgO, of mesoporous MgO and of MgO obtained from different precursors. Nevertheless, most of the AMS-doped MgO sorbents exhibit a CO_2_ uptake capacity of less than 50% of the maximum theoretical capacity. Table 1 shows different studies found in the literature using unsupported and supported MgO-based sorbents doped with AMS and the corresponding CO_2_ uptake capacity after multicyclic tests.

As mentioned above, the existence of a molten state generated by the melting of alkali metal salts is essential to increase the sorbent’s CO_2_ uptake and to improve the kinetic of MgO carbonation. In this work, the role of the NaNO_3_ single salt and the amount of a ternary alkali salt (NaNO_3_, LiNO_3_ and KNO_3_ (18/30/52; % mol)) on the CO_2_ uptake temperature range, as well as on the CO_2_ maximum uptake, will be evaluated for unsupported and supported sorbents. To evaluate the calcination atmosphere effect on the working temperature range of the alkali salts, its decomposition was studied under air and CO_2_ atmosphere. As innovative work in relation to the experiments shown in Table 1, the sorbent calcination will be performed under a CO_2_ atmosphere to obtain a concentrated CO_2_ stream useful for storage or utilization, which is more interesting for industrial applications.

## 2. Experimental Procedure

### 2.1. Materials

All the MgO-based sorbents were prepared with magnesium nitrate hexahydrate (Mg(NO_3_)_2_·6H_2_O), with an assay of 98–102% (Sigma-Aldrich, St. Louis, MO, USA), and for the supported materials were used calcium nitrate tetrahydrate (Ca(NO_3_)_2_·4H_2_O) or cerium nitrate hexahydrate (Ce(NO_3_)_3_·6H_2_O), both with an assay ≥ 99% (Sigma-Aldrich, St. Louis, MO, USA). The citric acid monohydrate (C_6_H_8_O_7_·H_2_O) with an assay of 99.5–102%, was used as a chelating agent (Panreac, Castelar del Vallés, Barcelona, Spain).

### 2.2. MgO-Based Sorbents Synthesis and Doping with Alkali Metal Salts

The Mg(NO_3_)_2_·6H_2_O was dissolved in distilled water with a molar ratio of citric acid and distilled water to magnesium of 1:1 and 120:1, respectively. For the supported MgO sorbents, the same procedure was used but adding Ca(NO_3_)_2_·4H_2_O or Ce(NO_3_)_3_·6H_2_O.

**Figure 1:**
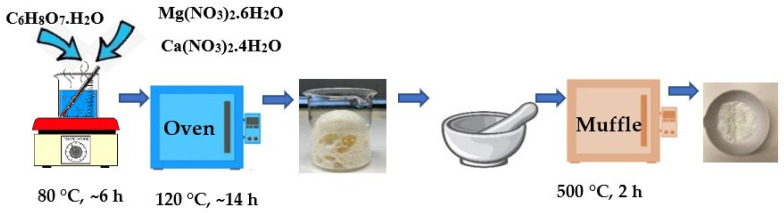
Synthesis of MgO based sorbents by the sol–gel method.

As shown in Figure 1, each solution was continuously stirred at 80 °C for 6 h. Afterward, the wet gel was dried in the oven at 120 °C for 14 h, milled, and the sample obtained was calcined in the muffle by ramping the temperature to 500 °C at a rate of 2 °C min^−1^ plus 2 h more at 500 °C.

The synthesized MgO-based sorbents were doped with mono AMS (NaNO_3_) and with a ternary AMS mixture (NaNO_3_, LiNO_3_ and KNO_3_ (18/30/52; mol.%)) [11,22] using the wet-impregnation method. Prior to the impregnation, the AMS was dried in the oven at 120 °C for 24 h. First, the MgO-based sorbent and AMS have been dissolved in 20 mL of distillate water, using magnetic stirring for 1 h at room temperature. The obtained aqueous slurry was dried in the oven at 120 °C for 14 h. Afterward, the dried sample was placed in the muffle for calcination using a heating rate of 2 °C min^−1^ until it reached 450 °C, plus 4 h at 450 °C. Table 2 summarizes all the prepared AMS-MgO-based sorbents and the different ratios of AMS in each sorbent. The following nomenclature was used for the doped sorbent samples: x (Na or Na-Li-K)-MgO-Z, where x corresponds to the molar fraction (%) of AMS, and Z corresponds to the Ca or Ce support.

### 2.3. Characterization Methods

A D8 Advance diffractometer (Bruker AXS GmbH, Karlsruhe, Germany) using Cu Kα (λ = 0.15406) radiation and a Ni filter was used to identify the crystalline phases of the synthetized sorbents before and after the cyclic experiments on a thermogravimetric analyzer (Setsys Evolution TGA, Setaram Instruments, Caluire, France). The equipment was operated at 30 mA and 40 kV, scanned within the 2θ range of 5–80°, and had a step size (scanning duration) of 0.03° (0.5 s). The crystallography open database (COD) was used to identify the crystalline phases. The MgO average crystallite size of the sorbents was estimated using Scherrer’s equation (D=Kλ/bcos⁡θ), based on the XRD data, where D is the crystallite size (nm), b corresponds to the full width at half maximum (FWHM) of the XRD peak considered, λ is the wavelength (0.15406 nm), θ is the Bragg angle (degree) and K is Scherrer’s constant (K = 0.9, assuming that particles are spherical).

The specific surface area (S_BET_) of the synthetized sorbents based on the BET method and the total pore volume (V_p_), were determined by the N_2_ sorption technique at −196 °C, (Quantachrome Instruments, Model autosorb IQ, Graz, Austria) at a relative pressure (p/p_0_) of 0.95. The degasification procedure was performed in two steps: the first one at 90 °C for 1 h and the second one at 350 °C for 5 h.

### 2.4. Thermal Decomposition of Mono and Ternary Alkali Nitrates Salts

The thermal decomposition of the molten salts was assessed for the pure NaNO_3_ and for the ternary mixture of 52% (mol) KNO_3_, 18% (mol) NaNO_3_ and 30% (mol) LiNO_3_ by thermogravimetric analysis with air and pure CO_2_ atmospheres between 25 and 1000 °C at a heating rate of 10 °C min^−1^.

### 2.5. CO_2_ Uptake TGA Studies

#### 2.5.1. Effect of Mono and Ternary AMS Molar Fraction and Inert Support Addition

The effect of mono or ternary alkali metal salts doping, as well as the best molar fraction of AMS-MgO were evaluated in a thermogravimetric analyzer (TGA) system TG-DSC Setsys Evo 16. The TGA studies were carried out with MgO-based sorbents for the range of temperature from 20 to 500 °C and a temperature ramp of 10 °C min^−1^. During the experiments, a constant flow of 80 mL min^−1^ of CO_2_ and ~10 mg of sample were used. The best two mono and ternary AMS samples, i.e., with higher CO_2_ uptake capacity, were identified and selected to proceed to the tests with five carbonation–calcination cycles in the TGA as described below. Then, using the same procedure, the effect of the addition of an inert support to the MgO sorbent was tested with Ca and Ce additions (10%, mol), considering the most promising AMS doping and the respective molar fraction.

#### 2.5.2. Carbonation–Calcination Cycles

A TGA system TG-DSC Setsys Evo 16 was used to carry out the studies for the assessment of both the cyclic stability and the CO_2_ uptake of the supported and unsupported MgO sorbents. These tests consisted of five carbonation–calcination cycles with carbonation and calcination temperatures of 300 °C (60 min) and 445 °C (60 min), respectively, using a constant flow of 80 mL min^−1^ of CO_2_, a ramp temperature of 10 °C min^−1^ and ~10 mg of sample. A blank experiment was performed to correct apparatus buoyancy effects like atmosphere density changes with temperature. The unsupported sample showing the best performance was identified and tested through 12 carbonation–calcination cycles using the same experimental conditions.

The TGA mass variation allows determining the mass of CO_2_ captured by the sorbent during the carbonation step (Equation (2)), and subsequently, the carrying capacity and the MgO conversion to carbonate can be obtained using Equations (3), (4) and (5), respectively.

(2) $$m_{{CO}_{2}captured}=m_{carbonation}-m_{calcination}$$

where $\;m_{CO2captured}$ is the mass of CO_2_ captured in each cycle, $m_{carbonation}$ is the mass of the sample after the carbonation reaction, and $m_{calcination}$ is the mass of the sample after the calcination reaction. The carrying capacity (CC) of the sorbent is defined as the mass of CO_2_ captured per mass unit of the sorbent (mg CO_2_ g^−1^ sorbent).

(3) $$CC=m_{{CO}_{2}captured}/m_{sorbent}$$

where $m_{sorbent}$ is the mass of the sorbent. The theoretical carrying capacity (${CC}_{theor.}$) is calculated with Equation (4),

(4) $${CC}_{theor.}=M_{{CO}_{2}}/M_{MgO}=1.09$$

where $M_{{CO}_{2}}$ and $M_{MgO}$ represent the molar mass of CO_2_ and MgO (g mol^−1^), respectively. The MgO conversion of the sorbent is defined as the percentage of the CC when compared to the ${CC}_{theor.}\;$

(5) $${MgO}_{conversion}=CC/\left({CC}_{theor.}\times w_{{MgO}_{sorbent}}\right)\times 100\left(\%\right)$$

where $w_{{MgO}_{sorbent}}$ represents the nominal mass fraction of the MgO (%, wt.) in the sorbent.

## 3. Results

### 3.1. Properties of MgO-Based Sorbents

**Figure 2:**
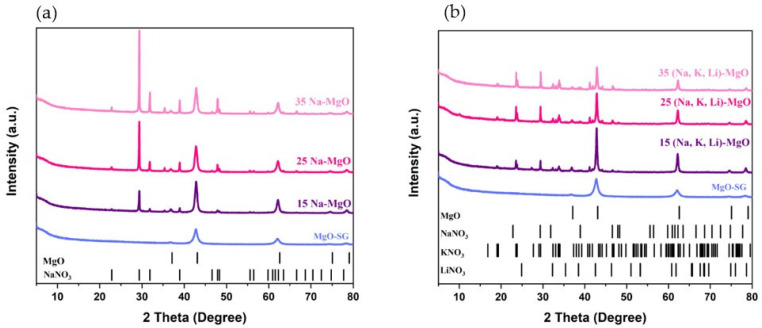
XRD patterns of MgO-SG sorbent doped with 15, 25 and 35 (mol, %) of: (**a**) NaNO_3_, (**b**) ternary mixture of KNO_3_, NaNO_3_ and LiNO_3_.

The crystalline structures of the MgO-SG sorbent impregnated with 15, 25 and 35% of either NaNO_3_ (Figure 2a) or a ternary mixture of KNO_3_, NaNO_3_ and LiNO_3_ (Figure 2b) were analyzed by powder XRD. All the XRD-obtained patterns show the characteristic peaks of the MgO (42.9°, 62.2°, 78.5°) pattern and a main peak located at ~29.4° that is referred to the NaNO_3_ pattern. For the case of the sorbents impregnated with the ternary alkali metals salts, the main peak of KNO_3_ (~23.6°) was also observed, and the second main peak should be overlaid by the NaNO_3_ peak (29.4°). The LiNO_3_ was not identified, which can be justified by its high dispersion on the sorbent sample, leading to the Li concentration below the instrumental detection limit [23] or its partial decomposition to Li_2_O_2_ or LiO_2_ whose main peaks are around 32.9° and 33.5°. Wang et al. [24] studied a similar ternary system, LiNO_3_ (25.9 mol.%), NaNO_3_ (20.06 mol.%) and KNO_3_ (54.1 mol.%), and concluded that unlike the sodium and potassium nitrate phases, lithium nitrate is an unstable phase in the ternary system that starts to decompose to Li_2_O_2_ and LiO_2_ at temperatures higher than 440 °C.

The MgO peaks are significantly sharper in the impregnated samples, suggesting that the impregnation influences the average size of MgO crystallite. It is usually expected that the sorbents with smaller MgO crystallites have higher surface areas and an enhanced reactivity; thus, the effect of impregnation on the growth of MgO crystallites is evaluated and correlated with the sorbent's performance along the carbonation–calcination cycles in Section 3.3. For instance, the average crystallite size of the MgO-SG sample is 10 nm, while the 15 Na-MgO presents a crystallite with 15 nm, and the MgO-SG impregnated with 15% of (Na, Li, K)NO_3_ achieves a crystallite size of 26 nm. Such results indicate that the impregnation with alkali metal nitrates boosts the growth of the MgO crystals, which is more pronounced in the case of the impregnation with the ternary mixture. On the other hand, the AMS molar percentage of the impregnation does not seem to influence significantly the growth of the MgO crystallite. The MgO-SG sorbent doped with NaNO_3_ shows an average crystallite size of ca. 15 nm and the MgO-SG sorbent doped with (Na, Li, K)NO_3_ of ca. 28 nm regardless the molar percentage of AMS impregnation (Table 3).

**Figure 3:**
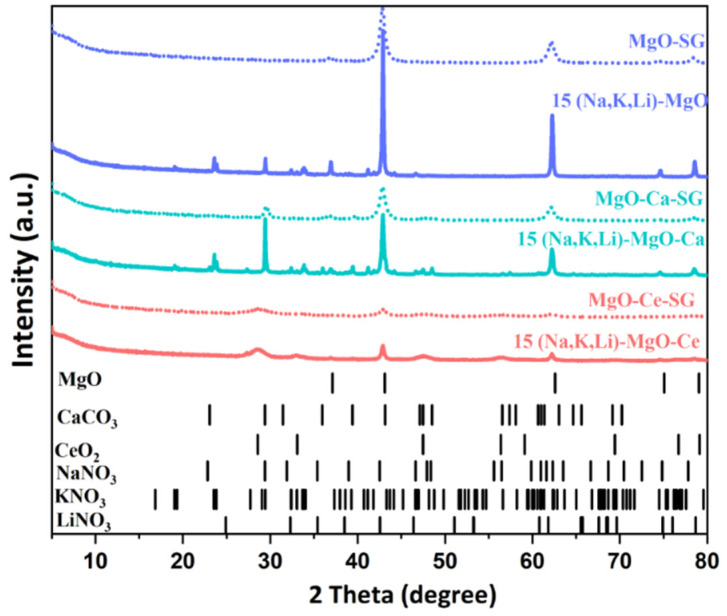
XRD patterns of MgO-SG, MgO-SG-Ca and MgO-SG-Ce before and after doping with 15(Na, K, Li)NO_3_.

The supported sorbents, MgO-Ca-SG and MgO-Ce-SG, were doped with 15% of a ternary mixture of (Na, Li, K)NO_3_. Figure 3 shows the *X*-ray diffraction peaks and Table 4 compares the MgO crystallite size for the unsupported and supported sorbents before and after the AMS doping.

The 15 (Na, Li, K)-MgO, 15 (Na, Li, K)-MgO-Ca and 15 (Na, Li, K)-MgO-Ce sorbents exhibit the characteristic peaks of the MgO pattern (42.9°, 62.2°, 78.5°). Moreover, the 15 (Na, Li, K)-MgO-Ca and 15 (Na, Li, K)-MgO-Ce also present the main peaks of the XRD pattern of CaCO_3_ (29.4°) and CeO_2_ (28.5°), respectively. Regarding the nitrate salts used in the impregnation, as justified above, the LiNO_3_ was not detected by XRD. The main peaks of the KNO_3_ pattern (23.5° and 29.4°) were identified for the 15 (Na, Li, K)-MgO and of the 15 (Na, Li, K)-MgO-Ca as well as the main peaks of the NaNO_3_ pattern (29.4°, 31.9°, 38.9°, 47.9°). However, for the 15 (Na, Li, K)-MgO-Ce sorbent, as the characteristic peak of the CeO_2_ XRD pattern is very wide, it is impossible to identify the peak at 29.4° of the KNO_3_ and NaNO_3_ patterns. Still, a small peak at 27° of the KNO_3_ pattern is exhibited as well as the peaks at 31.9°, 38.9°, 47.9° related to the NaNO_3_ pattern.

Table 4 shows that the AMS doping influenced the crystallite size of supported sorbents, causing it to increase but in a similar way for both supports.

**Figure 4:**
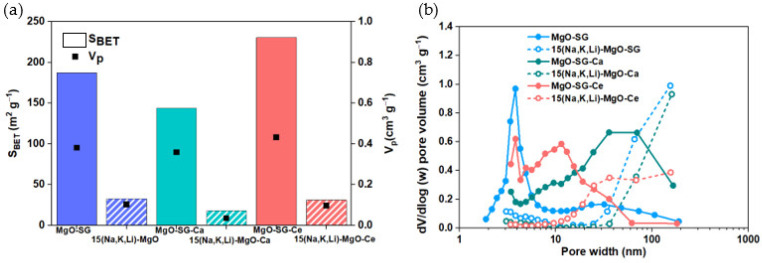
Textural properties of MgO-SG, MgO-SG-Ca and MgO-SG-Ce before and after doping with 15(Na, K, Li)NO_3_: (**a**) specific surface area (S_BET_) and total pore volume (V_p_), (**b**) pore size distribution (PSD) estimated by the BJH desorption.

The specific surface area (S_BET_) and the pore size distribution of MgO-SG, MgO-SG-Ca and MgO-SG-Ce before and after doping with 15 of (Na, K, Li)NO_3_ was evaluated (Figure 4a,b) by the N_2_ sorption technique.

The MgO-SG-Ce shows the highest S_BET_ (230 m^2^ g^−1^) followed by MgO-SG (187 m^2^ g^−1^) and the MgO-Ca-SG (144 m^2^ g^−1^). However, after doping, all the sorbents show a severe reduction in their S_BET_, namely, 83%, 88% and 87% for 15 (Na, Li, K)-MgO, 15 (Na, Li, K)-MgO-Ca and 15 (Na, Li, K)-MgO-Ce sorbents, respectively. This evidence is in line with the previous conclusions from the XRD characterization, in which we observed an increase in the MgO crystallite size, indicating a reduced surface area available for the MgO sorbent to uptake CO_2_.

Dal Pozzo et al. [11] reported a S_BET_ of 22 m^2^ g^−1^ for a MgO sorbent promoted with 10% of (Na, Li, K)NO_3_, which is smaller than that of 15 (Na, Li, K)-MgO and 15 (Na, Li, K)-MgO-Ce sorbents but higher than that of 15 (Na, Li, K)-MgO-Ca. In addition, all the analyzed sorbents of the set also registered a decrease in the total pore volume after AMS doping. This is probably due to its partial occupation by the ternary mixture [17], which should affect the CO_2_ uptake due to the higher occurrence of pores’ blocking.

The PSD of the MgO-SG and MgO-SG-Ce sorbents (Figure 4b) indicates a predominance of the presence of mesopores (2–50 nm). There is also a small percentage of macropores (>50 nm). The MgO-SG-Ca consists mainly of a macroporous sorbent, although the MgO-SG-Ca also exhibits a small share of mesopores. After doping with 15(Na, K, Li)NO_3_, all the sorbents are predominantly made up of macropores (>50 nm), but in case of 15 (Na, Li, K)-MgO-Ce, a small share of mesopores is still present.

### 3.2. Thermal Decomposition of Mono and Ternary Alkali Nitrates Salts

**Figure 5:**
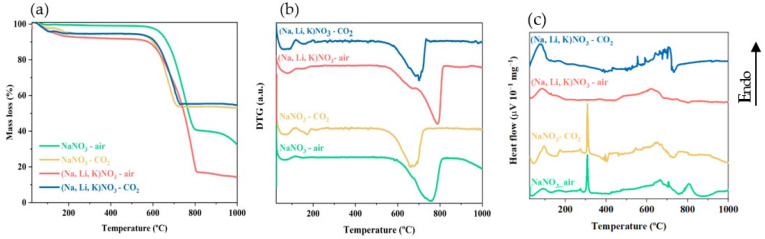
Thermogravimetric analysis: (**a**) thermal decomposition in mass loss (%), (**b**) first derivative of mass change and (**c**) heat flow profiles of NaNO_3_ and (Na, Li, K)NO_3_ with temperature.

The mass loss (%), the first derivative of the mass change (DTG) and the heat flow profiles with temperature were obtained in the TGA, between 25 and 1000 °C (heating rate: 10 °C min^−1^) under 100% of air or 100% of CO_2_ flow, for pure NaNO_3_ and a ternary mixture of 52 mol.% KNO_3_, 18 mol.% NaNO_3_ and 30 mol.% LiNO_3_ (Figure 5a–c). The knowledge about the mono and ternary alkali nitrates thermal decomposition under different atmospheres contributes to the understanding of the AMS stability on the doped sorbents.

Figure 5a shows a mass loss until 200 °C related with the release of the adsorbed water, since these salts are very hygroscopic [25]. It stands out that all the samples remain almost stable up to a temperature of around 600 °C, where an abrupt loss of mass is observed regardless of the atmosphere. The atmosphere affects the extent of the salt’s decomposition: under pure air atmosphere, higher decomposition temperatures (~800 °C) are achieved when compared to those achieved for the case of 100% CO_2_ atmosphere (~715 °C) [26]. Due to the formation of alkali carbonates with higher molar mass than the corresponding oxides, i.e., Na_2_O, K_2_O and Li_2_O/Li_2_O_2_, the observed mass loss is lower under a CO_2_ atmosphere. The alkali carbonates are very stable at high temperature, especially under CO_2_ atmosphere; i.e., Na_2_CO_3_ and K_2_CO_3_ start to decompose slowly above 900 °C [27] and, in the case of Li_2_CO_3_, the decomposition starts at 737 °C, but only at temperatures near 900 °C is the decomposition significant [26].

The decomposition temperature of each sample can be defined as the upper stability limit of a mixture (T3): that is, the maximum temperature at which it loses 3% of the initial weight [25]. The definition of the decomposition temperature allowed to set the working temperature range with the melting point and the T3 being the extremes of that interval [25]. In the present study, since we identified the presence of water in all the samples, the initial weight was replaced by the weight at 200 °C. The decomposition temperature range was also determined. The results obtained are summarized in Table 5.

The results show that the ternary mixture offers a larger temperature window to operate when compared with the single salt, NaNO_3_. This advantage relies mostly on that already described in the literature: the addition of the LiNO_3_, which significantly decreases the melting temperature of the mixture with relation to the sodium nitrate salt, that is, 120 °C [28] against 308 °C [11], and not in the T3, which do not vary as much between both cases. Nevertheless, in air atmosphere, the eutectic mixture presents a T3 lower than the single salt, which is associated with the presence of Li that prematurely decomposes as stated by Bauer [28], but it does not happen under the CO_2_ flow, evidencing that the atmosphere type is also a relevant parameter.

The heat flow TG curves presented in Figure 5c exhibit several up and downward peaks, whether an endothermic or exothermic heat event occurs, respectively, which allow properly identifying that the temperature occurs at each event. The analysis of both the heat flow profile (Figure 5c) and corresponding DTG curves (Figure 5b) reveals the existence of corresponding peaks in the same temperature intervals for all the samples (T3 ≥ 575 °C) regardless of the atmosphere considered. In addition, it can be observed that the NaNO_3_ melting (endothermic reaction) occurs around 310 °C (Figure 5c), which agrees with the literature [28]. In the case of ternary salt, it was difficult to identify its melting. Due to the water vaporization, which was confirmed by the mass loss in both salts, only a small peak around 129 °C was visible for (Na, Li, K)NO_3_ salt, meaning that probably the water vaporization is masking the endothermic reaction associated with the ternary salt melting.

### 3.3. CO_2_ Uptake TGA Studies

#### 3.3.1. Assessment of the Effect of Mono and Ternary AMS Molar Fraction

**Figure 6:**
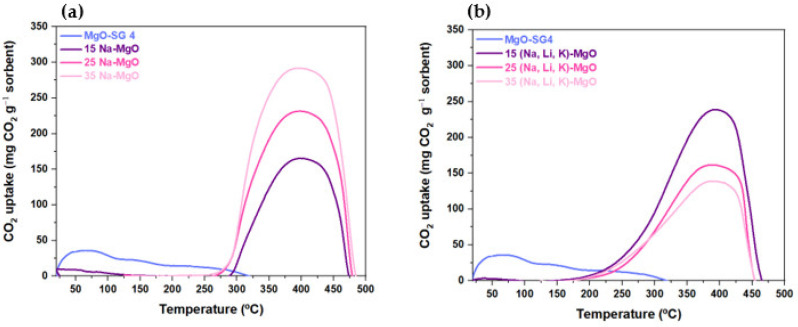
Profile of CO_2_ uptake of undoped MgO-SG sorbent and doped with 15%, 25% and 35% of (**a**) NaNO_3_ or (**b**) (Na, Li, K)NO_3_ performed under a 100% CO_2_ atmosphere.

Temperature-programmed carbonation–calcination followed by TGA studies were carried out with samples of MgO sorbent undoped and doped with different molar fractions of mono or ternary AMS to assess the effect of the promotors ratio (AMS/MgO) on the CO_2_ uptake (Figure 6a,b).

Undoped MgO-SG sorbent immediately captures CO_2_ in the beginning of the TGA test, achieving its maximum CO_2_ uptake at 50 °C, and gradually releases it with the increasing of the temperature from 75 to 325 °C. The corresponding CO_2_ desorption peak is significantly wider than those of the MgO-doped samples, which is probably due to the type of surface of each sorbent and, in turn, with the type of species being formed during the CO_2_ uptake. In contrast, the extent of CO_2_ uptake is insignificant for all the doped MgO-SG sorbents at low temperatures until a certain value of temperature is reached, where it starts to uptake CO_2_ significantly. According to the literature, in the case of the undoped MgO-SG sorbent, the CO_2_ molecules were quickly adsorbed at the surface of the MgO particles to form an unidentate carbonate layer impermeable for gaseous reactants. On the other hand, in nitrate-promoted MgO, the molten layer dissolves the CO_2_. Since the diffusivities of the carbonate ions and the oxygen ions in MgCO_3_ layers are higher in the absence of the unidentate layers, the formation of MgCO_3_ occurs faster [11]. This enhancement is justified by the melting of the nitrates, since the CO_2_ solubility increases in the phase transition from the solid to liquid state of the nitrates. In this way, the AMS impregnation is proved to increase considerably the CO_2_ uptake by the MgO sorbents. The mentioned temperature was determined as the inflection point of each CO_2_ uptake curve, that is, where the corresponding second derivate changes its signal. The different threshold temperatures were obtained for each sorbent (Supplementary Materials, Figure S1), and the results are summarized in Table 6. The sharp increase in the CO_2_ uptake by the doped sorbents, with the temperature, can be attributed to the physical state of the corresponding AMS [29], ranging from solid to melting. At low temperatures, the CO_2_ uptake is hindered due to the formation of a solid layer of nitrates that covers the surface of the sorbent. Only with the increasing temperature until close to the melting point of the AMS, this covering solid layer starts to melt. It is called the pre-melting phenomenon, where an interfacial liquidlike film of the partially disordered surface of the promoted sorbent is formed at temperatures below the corresponding melting point, enhancing the kinetic of the CO_2_ uptake [29]. For instance, the melting point of the single salt NaNO_3_ is 308 °C, but it suffers a solid-state transition from an ordered to a disordered rhombohedral structure at a lower temperature of 275 °C that, in turn, is believed to potentiate its pre-melting [11]. The present experiments indicate that this transition occurs at even lower temperatures (234–240 °C). In addition, the MgO-SG sorbent doped with single NaNO_3_ appears to have a higher threshold temperature than the corresponding MgO-SG sorbent doped with the ternary mixture. Hence, it is suggested that the eutectic ternary mixture lowers the CO_2_ uptake temperature. One study in the literature [11] describes a CO_2_ uptake temperature of 160 °C using the ternary eutectic mixture of (Na, Li, K)NO_3_. In this work, it was possible to reduce the temperature to around 175 °C. The difference between the inflection temperature and the temperature that corresponds to the maximum CO_2_ uptake (mg CO_2_ g^−1^ sorbent) achieved during the temperature-programmed experiment suggests that at low temperatures, the accelerating effect of the addition of alkali metal nitrates on the CO_2_ uptake by MgO is reduced, which is justified by the thermodynamic equilibrium of MgO/MgCO_3_ [11].

The maximum instantaneous rate of change in temperature with respect to CO_2_ uptake was obtained based on the upward peak of the first derivative of each TG curve of the tested sorbents (Supplementary Materials, Figure S1). The results indicate that the maximum CO_2_ uptake rate temperature does not significantly vary with the AMS impregnation percentage for both sorbents (Table 6). The samples of MgO sorbent impregnated with the ternary mixture show lower maximum conversion temperatures than those impregnated with the single salt. As the former presents lower inflection temperatures, it is also possible to associate them with a larger CO_2_ uptake temperature range, which extends the carbonation region. Moreover, the highest CO_2_ uptake of 292 mg CO_2_ g^−1^ sorbent was obtained for the MgO-SG sorbent doped with 35% of NaNO_3_, which, in turn, corresponds to the highest percentage of NaNO_3_. This result suggests that the sorbents with higher percentages of NaNO_3_ might favor the Mg^2+^ ions diffusion on the molten NaNO_3_ [11]. For the ternary mixture, the highest CO_2_ uptake was 239 mg CO_2_ g^−1^ sorbent for the MgO-SG doped with 15% of AMS and decreased with the increasing AMS molar fraction. This suggests that a certain thickness of the molten layer covering the surface of the sorbent hinders the CO_2_ uptake by increasing the mass transfer resistance. For the synthesized undoped MgO sorbent, there was found to be a maximum CO_2_ uptake of 36 mg CO_2_ g^−1^ sorbent, which is better than the value found in the literature (19 mg CO_2_ g^−1^ sorbent) [11].

**Figure 7:**
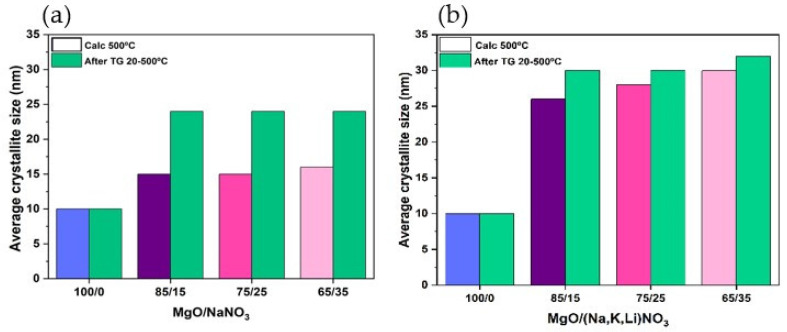
Crystallite size of undoped MgO-SG sorbent and doped with 15%, 25% and 35% of (**a**) NaNO_3_ or (**b**) (Na, Li, K)NO_3_, before (blue, violet, dark pink and light pink bars) and after (green bars) TG test between 20 and 500 °C.

Figure 7a,b shows the variation of the MgO crystallite size (CS) of the sorbents before and after the TG tests. Summarizing, the AMS impregnation contributes to the growth of the MgO crystallites with a higher increase in the MgO crystallite size when the impregnation is carried out using the ternary mixture of KNO_3_, NaNO_3_ and LiNO_3_ than the single salt NaNO_3_. The variation of the MgO crystallite size $(∆CS={CS}_{dopedsorb}-{CS}_{undopedsorb}/{CS}_{undopedsorb}\times 100$) of the sorbent doped with NaNO_3_ varies between 50 and 60%, but it increases for values between 160 and 200% for the sorbent with higher (Na, Li, K)NO_3_ content. Conversely, the samples impregnated with NaNO_3_ have a greater increase in the crystallite size before and after the TGA tests, i.e., between 50 and 60%, and 7% to 15%, for NaNO_3_ and (Na, Li, K)NO_3_, respectively. The AMS molar percentage of the impregnation does not affect the crystallite size regardless of using the single salt or the ternary mixture.

**Figure 8:**
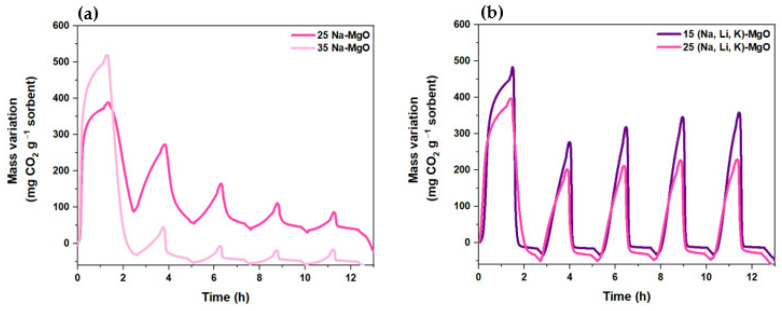
Profile of carbonation (300 °C, 60 min) and calcination (445 °C, 60 min) tests carried out along 5 cycles in a TG (80 mL min^−1^ of CO_2_) for (**a**) 25% and 35% of NaNO_3_ or (**b**) 15% and 25% (Na, Li, K)NO_3_ impregnated MgO-SG sorbent.

**Figure 9:**
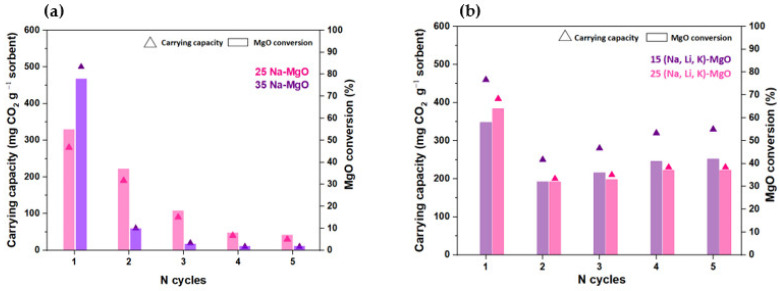
Carrying capacity (mg CO_2_ g^−1^ sorbent) and MgO conversion (%) of (**a**) 25% and 35% of NaNO_3_ or (**b**) 15% and 25% (Na, Li, K)NO_3_ impregnated sorbents achieved along 5 carbonation–calcination cycles.

The CO_2_ uptake and the cyclic stability of the most promising doped sorbents identified above for both AMS doping, i.e., 25% and 35% for NaNO_3_ and 15% and 25% of (Na, Li, K)NO_3_, were tested in a TGA by performing five carbonation–calcination cycles. Figure 8a,b show the cyclic CO_2_ carbonation–calcination profiles obtained in the TGA apparatus and Figure 9a,b show the respective carrying capacity and MgO conversion obtained for the MgO-SG sorbent impregnated with the single salt NaNO_3_ (25 and 35%, mol) and with the ternary mixture of (Na, Li, K)NO_3_ (15 and 25%, mol).

**Figure 10:**
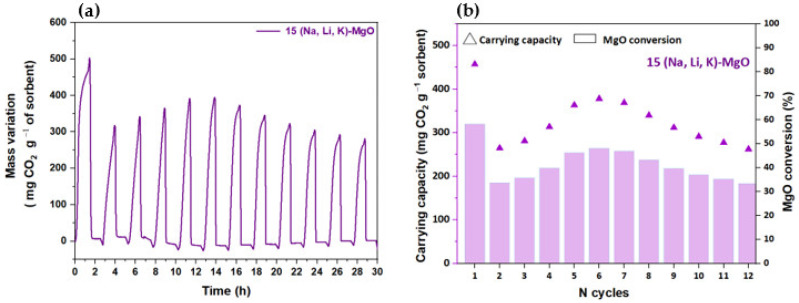
MgO-SG sorbent impregnated with 15% of (Na, Li, K)NO_3_: (**a**) profile of carbonation (300 °C, 60 min) and calcination (445 °C, 60 min) and (**b**) carrying capacity (mg CO_2_ g^−1^ sorbent) and MgO conversion (%) for tests carried out along 12 cycles in a TG (80 mL min^−1^ of CO_2_).

Both MgO-SG sorbents impregnated with 35% of NaNO_3_ and with 25% of NaNO_3_ exhibit a sharp decay on the CO_2_ uptake in the 2nd cycle followed by a gradual reduction until the 5th cycle. The 25 Na-MgO registered an MgO conversion of 55% in the 1st cycle, of 35% in the 2nd cycle and of only 7% in the last cycle. The 35 Na-MgO achieved even higher reductions with an MgO conversion of 78% in the 1st cycle, 10% in the 2nd cycle and only 2% in the 5th cycle. A qualitative comparison of Figure 8a,b immediately reveals that the MgO-SG samples impregnated with the ternary mixture of (Na, Li, K)NO_3_ have a better performance than those impregnated with the single salt NaNO_3_. Regardless of the molar percentage of ternary impregnation used, the two tested samples exhibited consistent peaks after five carbonation–calcination cycles, showing good CO_2_ uptakes and cyclic stability. The 15 (Na, Li, K)-MgO reached a MgO conversion of 58% and of 42% in the 1st and 5th cycles, respectively. Meanwhile, the 25 (Na, Li, K)-MgO presented a MgO conversion of 64% in the 1st cycle and of 37% in the last cycle. Although the 25 (Na, Li, K)-MgO has the highest MgO conversion in the 1st cycle, it shows the lowest MgO conversion in the last cycle. Hence, between all the studied sorbents, the 15 (Na, Li, K)-MgO sorbent was considered the most promising since the MgO conversion values remained stable over five carbonation–calcination cycles. Subsequently, a TG test was carried out with 12 carbonation–calcination cycles using the 15 (Na, Li, K)-MgO sorbent (Figure 10a). Figure 10b plots the carrying capacity and the MgO conversion of the cyclic CO_2_ carbonation–calcination test of the 15 (Na, Li, K)-MgO sorbent.

The literature [11] reports a study with a MgO sorbent doped with 10% of (Na, Li, K)NO_3_ that registered carrying capacities of 480 and 410 mg CO_2_ g^−1^ sorbent for the 1st and 5th cycles, respectively. Another article describes the carrying capacities of 440 and 352 mg CO_2_ g^−1^ sorbent for the 1st and 5th cycles, respectively, of a MgO sorbent impregnated with 15% of the same ternary mixture [19]. Since the 15 (Na, Li, K)-MgO sorbent synthesized in this work presents a carrying capacity between 460 and 330 mg CO_2_ g^−1^ sorbent from the 1st to the 5th cycle, it is clear that these results are in agreement with the results found in the literature. Dal Pozzo et al. [11] justify the MgO-based sorbents’ deactivation along cycles with the nitrate’s segregation. As shown in Figure 5a,b, the NaNO_3_ and (Na, Li, K)NO_3_ decomposition only starts above 600 °C, so these sorbents remain stable under the temperatures used for the above carbonation–calcination tests. However, since the AMS melting starts at lower temperatures (Table 5 and Figure 5c), the segregation of AMS can occur as it was demonstrated by SEM images for NaNO_3_ [11] and in our previous work (unpublished), where the segregation of K particles was also demonstrated.

Due to the AMS segregation, the dispersion of MgO in the melted phase of the nitrates will be spoiled, and the CO_2_ carrying capacity decreases. Apparently, under the used conditions, since the CO_2_ uptake during the 1st carbonation is more effective and faster (the CO_2_ uptake temperature range is lower) for the NaNO_3_ salt, the nitrates segregation is more pronounced, and the sorbent’s deactivation after the 1st cycle is abrupt.

In general, the 15 (Na, Li, K)-MgO sorbent exhibited a stable carbonation–calcination profile over the 12 cycles. Its MgO conversion dropped from 58% to 34% from the 1st to the 2nd cycle, but it gradually increased up to 48% until the 6th cycle, from where it progressively decreased until the last cycle, reaching a final MgO conversion of 33%. The decrease in MgO conversion is more significant after the 1st cycle, which can be correlated with the fast initial MgO-CO_2_ carbonation reaction, increasing the MgCO_3_ content in the sorbent and contributing to the alkali’s segregation [11]. Indeed, due to the segregation, the alkali will be less dispersed on the MgO particles, and its role as a phase transfer catalyst between bulk MgO and CO_2_ molecules decreases alongside the CO_2_ capture capacity. Thus, between the 2nd and the 6th cycles, the carbonation reaction occurred slowly, and more time was required for the carbonation stabilization as confirmed by the narrow peaks shown in Figure 10a and Figure S2 in the Supplementary Materials. However, it seems that there is an arrangement involving the alkalis and the MgO that allows the sorbent to recover some CO_2_ carrying capacity. After the 7th cycle, the carbonation reached the stabilization faster (evidenced in Figure 10a and Figure S2 in the Supplementary Materials), meaning that the alkalis were still enhancing the kinetic stage of carbonation reaction, but the sorbent started to lose its capture capacity. Harada et al. [19] reported carrying capacities of 440 and 310 mg CO_2_ g^−1^ sorbent between the 1st and the 12th cycle for a MgO sorbent doped with 15% of (Na, Li, K)NO_3_. In line with the literature, the 15 (Na, Li, K)-MgO synthesized in this work achieved a carrying capacity of 460 mg CO_2_ g^−1^ sorbent in the 1st cycle and 262 mg CO_2_ g^−1^ sorbent in the last cycle.

#### 3.3.2. Assessment of the Effect of Inert Support Addition

**Figure 11:**
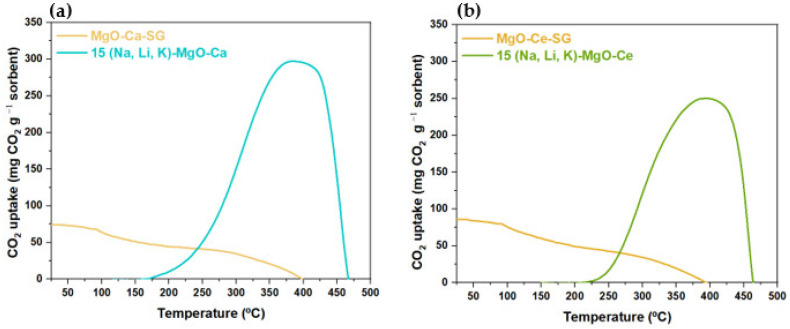
Profile of CO_2_ uptake of supported MgO sorbents undoped and doped with 15% of (Na, Li, K)NO_3_: (**a**) Ca-based support or (**b**) Ce-based support.

Temperature-programmed carbonation–calcination studies followed by TGA were carried out on samples of supported MgO sorbents (Ca or Ce-based) undoped and doped with 15% of (Na, Li, K)NO_3_ to assess the effect of the support on CO_2_ uptake (Figure 11a,b).

Undoped MgO-SG-Ca and MgO-SG-Ce sorbents have their maximum CO_2_ uptake of 73 mg CO_2_ g^−1^ sorbent and 86 mg CO_2_ g^−1^ sorbent, respectively, at the beginning of the TGA test at 25 °C, and we observed a gradual CO_2_ release with the increase in temperature until ~400 °C. Both supported sorbents exhibit CO_2_ calcination peaks significantly wider than those of the corresponding unsupported AMS sorbents (Figure 6). Conversely, the CO_2_ uptake of the 15 (Na, Li, K)-MgO-Ca and of the 15 (Na, Li, K)-MgO-Ce sorbents is negligible at low temperatures until it reaches the respective inflection temperature. This parameter was obtained as it was explained in Section 3.3.1, and the respective plots can be found in the Supplementary Materials, Figure S3. The maximum instantaneous rate of change in temperature with respect to CO_2_ uptake (°C) was also determined, as it was explained above. Both parameters are summarized in Table 7. The promoted MgO-SG-Ce sorbent showed a higher inflection temperature than the promoted MgO-SG-Ca, meaning that the latter starts the CO_2_ uptake sooner. As expected, the AMS doping enhanced considerably the CO_2_ uptake of both MgO sorbents, 15 (Na, Li, K)-MgO-Ca and 15 (Na, Li, K)-MgO-Ce sorbents achieving a maximum CO_2_ uptake of 299 mg CO_2_ g^−1^ sorbent and 252 mg CO_2_ g^−1^ sorbent at 384 °C and 393 °C, respectively. Summarizing, undoped MgO-SG-Ce exhibited higher CO_2_ uptake than MgO-SG-Ca, but AMS doping was more effective for the latter, with 15 (Na, Li, K)-MgO-Ca exhibiting higher CO_2_ uptake than 15 (Na, Li, K)-MgO-Ce. Moreover, 15 (Na, Li, K)-MgO-Ca also provides a more extensive carbonation than 15 (Na, Li, K)-MgO-Ce, since the former presents a wider carbonation temperature range than the latter.

Papalas et al. [20] report a CO_2_ uptake of 317 mg CO_2_ g^−1^ sorbent for an MgO sorbent promoted with 5% (mol) of CaCO_3_ and impregnated with 20% of ternary mixture (Na, Li, K)NO_3_ at 300 °C for 30 min under an atmosphere consisting of 30% of CO_2_, which is in line with the CO_2_ uptake obtained in the present work for the 15 (Na, Li, K)-MgO-Ca sorbent, despite the difference between the CO_2_ atmospheres. Jin et al. [21] synthesized a supported sorbent with CeO_2_ (10% mol) impregnated with a mixture of LiNO_3_:NaNO_3_:Na_2_CO_3_:K_2_CO_3_ = 0.2:0.76:0.04:0.5 (17% mol) MgO sorbent that achieved a CO_2_ uptake of 450 mg CO_2_ g^−1^ sorbent at 325 °C for 120 min under 100% CO_2_, which is almost double the CO_2_ uptake obtained for the 15 (Na, Li, K)-MgO-Ce. It is important to note that in the above example a temperature plateau of 120 min (325 °C) was applied, while the present study does not include a temperature plateau.

The 15 (Na, Li, K)-MgO-Ca sorbent starts to uptake CO_2_ at a lower temperature (153 °C) than the 15 (Na, Li, K)-MgO-Ce (210 °C). However, the last one reaches the maximum instantaneous rate of change in temperature with respect to CO_2_ uptake at a lower temperature (294 vs. 308 °C). The higher inflection temperature of 15 (Na, Li, K)-MgO-Ce was unexpected, since the same alkali ternary mixture was used. An explanation can be assigned to the high dispersion of both the CeO_2_ and AMS on the sorbent. As it can be observed in the XRD patterns of the samples supported with Ce (Figure 3), broader peaks are observed, meaning there is a high dispersion of these compounds in the sorbent, which probably delays the molten effect of the ternary alkali salts mixture on the CO_2_ uptake. Yu et al. [30] synthesized CeO_2_-MgO and MgO sorbents and found that despite the similarity of their initial surface areas, the doped sorbent had an increased CO_2_ uptake, which was justified by changes in the pore structures and by the increase in the basicity of the MgO phase induced by the addition of CeO_2_. In the present study, the corresponding values of S_BET_ are also similar (31 vs. 32 m^2^ g^−1^), and comparatively with the unsupported sorbent, the maximum CO_2_ uptake of 15 (Na, Li, K)-MgO-Ce (239 vs. 252 mg CO_2_ g^−1^ sorbent) was slightly improved. It must be noted that no temperature plateau was considered during this experiment. It is important to note that as shown in Table 7 and in accordance with the literature [2], the addition of both Ca and Ce-based supports improved the MgO sorbents’ performance.

**Figure 12:**
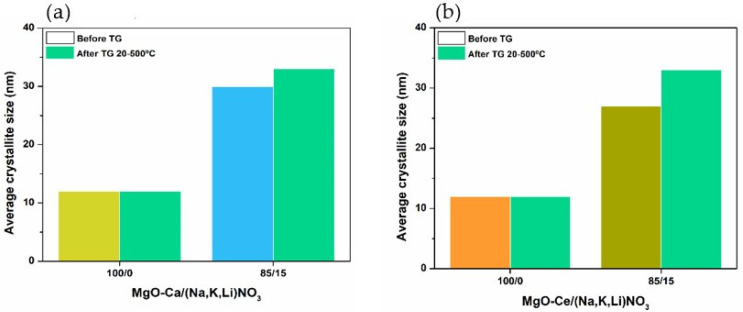
Average crystallite size of supported MgO sorbents undoped and doped with 15% of (Na, Li, K)NO_3_ before (yellow, blue, orange and mustard bars) and after (green bars) TG test between 20 and 500 °C: (**a**) Ca-based support or (**b**) Ce-based support.

Figure 12a,b shows the variation of the MgO crystallite size of the undoped and doped sorbents with the Ca- or Ce- based support before and after the TG tests.

Both undoped MgO-SG-Ca and MgO-SG-Ce sorbents maintained the MgO crystallite size after undergoing the TGA test. The same behavior was already observed for the unsupported sorbent (Figure 7). For 15 (Na, Li, K)-MgO-Ca and 15 (Na, Li, K)-MgO-Ce, the MgO crystallites size increased 150% and 125% (Table 4), which was mainly due to the AMS impregnation procedure, while after the TGA experiment, the increase was only 10% and 22%, respectively. Hence, the AMS impregnation with 15% (Na, Li, K)NO_3_ affected identically the supported MgO-Ca-SG and MgO-Ce-SG sorbents.

**Figure 13:**
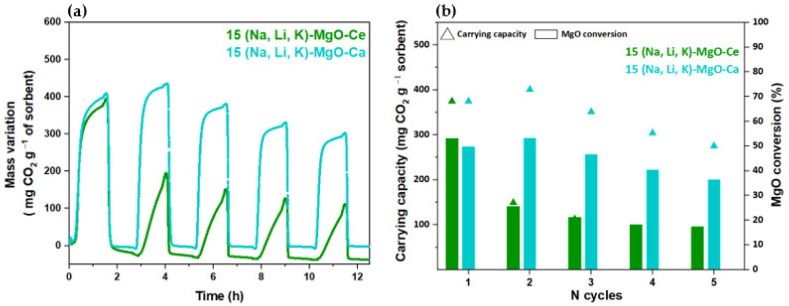
MgO-SG supported sorbents (Ca- or Ce- based support) impregnated with 15% of (Na, Li, K)NO_3_: (**a**) profile of carbonation (300 °C, 60 min) and calcination (445 °C, 60 min) and (**b**) carrying capacity (mg CO_2_ g^−1^ sorbent) and MgO conversion (%) for tests carried out along 12 cycles in a TG (80 mL min^−1^ of CO_2_).

The performance of supported sorbents along the cyclic carbonation–calcination cycles was assessed in TGA tests (Figure 13a,b).

The 15 (Na, Li, K)-MgO-Ca sorbent exhibited a more promising carbonation–calcination profile along the five cycles than the 15 (Na, Li, K)-MgO-Ce sorbent, since a higher CO_2_ carrying capacity was achieved over cycles. However, both supported sorbents present quite stable peaks until the end of the test. Quantitively, the MgO-SG-Ca impregnated with 15% of (Na, Li, K)NO_3_ obtained a carrying capacity of 375 mg CO_2_ g^−1^ sorbent in the 1st cycle and of 275 mg CO_2_ g^−1^ sorbent in the 5th cycle. Papalas et al. [20] reports a Ca-based supported MgO promoted with 20% of ternary mixture (Na, Li, K)NO_3_ that varied its carrying capacity from 396 to 330 mg CO_2_ g^−1^ sorbent for the same number of cycles, which is in agreement with the obtained results. Jin et al. [21] report a Ce-based supported MgO sorbent promoted with 12% of ternary mixture of (Na, Li, K)NO_3_ with carrying capacities of 340 and 180 mg CO_2_ g^−1^ sorbent between the 1st and the 5th cycles. The same sorbent but with an impregnation molar percentage of 17% managed to achieve 420 mg CO_2_ g^−1^ sorbent in the 1st cycle and 330 mg CO_2_ g^−1^ sorbent in the 5th cycle. The 15 (Na, Li, K)-MgO-Ce sorbent presents an impregnation molar percentage of 15%, which is in between those mentioned in the literature, and it achieved a carrying capacity of 375 mg CO_2_ g^−1^ sorbent, which is within the same range. In fact, the unsupported 15(Na, K, Li)-MgO sorbent performs better than the supported sorbents, since a higher carrying capacity was achieved for this sorbent, i.e., 460 and 330 mg CO_2_ g^−1^ sorbent for the 1st and 5th cycle, respectively. Nevertheless, after the 1st carbonation–calcination cycle, the 15 (Na, K, Li)-MgO-Ca is the unique one that maintains a similar carbonation rate profile along the cycles. The carbonation profiles (Figure 8 and Figure 13a) show that the 1st carbonation rate is similar for all the sorbents, but after the 2nd cycle, the carbonation slope decreases, meaning that the carbonation rate is delayed, except for (Na, K, Li)-MgO-Ca. The carbonation reaction kinetics is one of the MgO-based sorbent’s drawbacks, and the faster carbonation of 15 (Na, K, Li)-MgO-Ca is a huge advantage, which can contribute to a comparative upscale of MgO sorbents. Similar results were found by Cui et al. [31]; the authors performed kinetic studies with Ca and Mg precursors and verified that the activation enthalpy of CO_2_ sorption for doped AMS-MgO sorbent was lower than for the undoped AMS-MgO, indicating that the CaCO_3_ decreases the activation energy of CO_2_ sorption on MgO. The CaCO_3_ should act as a spacer among MgO crystallites, enhancing the AMS dispersion on the MgO surface area and making the carbonation reaction easier.

The positive effect of the calcium support was already evidenced by the lower inflection temperature of the 15 (Na, K, Li)-MgO-Ca sorbent relatively to 15 (Na, K, Li)-MgO (153 vs. 175 °C, Table 7) and the maximum CO_2_ uptake obtained when no temperature plateau was applied (299 vs. 239 mg CO_2_ g^−1^ sorbent, Table 7), respectively.

## 4. Conclusions

This study evaluated the role of a NaNO_3_ salt and a ternary mixture (NaNO_3_, LiNO_3_ and KNO_3_ (18/30/52; % mol.)) molar fraction (15, 25 and 35%) on the MgO carbonation temperature range and on the maximum CO_2_ uptake temperature. Regarding the carbonation temperature range, the ternary mixture offers a larger temperature window of operation when compared to that of the single salt NaNO_3_, which is justified by the lower melting temperature of the ternary mixture (120 vs. 308 °C). Also, it is observed that the increase in the percentage of the NaNO_3_ single salt between 15 and 35% allows enhancing the CO_2_ uptake by the sorbent, but for the case of the ternary mixture sorbents, the opposite behavior is observed. This should be explained by the lower melting temperature of the ternary mixture that promotes the enhancement of the AMS dispersion on the MgO surface at the selected carbonation temperature (300 °C). Probably, the increase in the thickness of the molten layer covering the surface of the sorbent beyond a certain limit hinders the CO_2_ uptake by increasing the mass transfer resistance, which justifies the enhanced results obtained with 15% of the ternary mixture. Despite the lower melting temperature of the ternary mixture, the inflection temperature only starts around 175 °C, suggesting that at low temperatures, the accelerating effect of the addition of the ternary alkali metal nitrates on the CO_2_ uptake by MgO is reduced, which is justified by the thermodynamic equilibrium of MgO/MgCO_3_.

Regarding the supported sorbents (Ca and Ce- based), both show a lower carrying capacity along the carbonation–calcination cycles than the unsupported sorbent. However, the 15 (Na, K, Li)-MgO-Ca sorbent shows a stable slope for the carbonation, while for the other sorbents, it decreases after the 1st cycle, meaning that the maximum carbonation rate was delayed. Thus, the carbonation performs faster for the 15 (Na, K, Li)-MgO-Ca sorbent, overcoming some kinetic limitations, which is considered as a huge advantage, and it can contribute for a sooner upscale of MgO sorbents for industrial CO_2_ capture applications.

## Figures and Tables

**Table 1:** CO_2_ uptake and experimental conditions of unsupported and supported MgO-based sorbents impregnated with AMS.

MgO-Based Sorbent	Temperature (°C)/Atmosphere/Time (min)		Number of Cycles	CO_2_ Uptake (mg CO_2_ g^−1^ Sorbent)		Ref.
	Carbonation	Calcination		Initial	Final	
Unsupported: xAMS—MgO						
10 NaNO_3—_MgO	300/10% CO_2_/180	300/He/180	5	390	210	[17]
10 NaNO_3—_MgO	300/CO_2_/30	450/N_2_/30	20	140	100	[18]
10 (Li, Na, K)NO_3—_MgO	300/CO_2_/60	450/N_2_/15	5	480	410	[11]
15 (Li, Na, K)NO_3—_MgO	300/CO_2_/60	350/N_2_/30	5	440	350	[19]
15 (Li, Na, K)NO_3—_MgO	300/CO_2_/60	350/N_2_/30	12	440	310	[19]
Supported: xAMS—MgO—Y support						
20 (Li, Na, K)NO_3—_MgO_—_5 Ca 12 (Li, Na, K)NO_3—_MgO_—_10 Ce 17 (Li, Na, K)NO_3—_MgO_—_10 Ce	300/CO_2_/30	425/N_2_/10	5	396	330	[20]
	300/CO_2_/60	425/N_2_/15	5	340	180	[21]
	300/CO_2_/60	425/N_2_/15	5	420	330	[21]

x—molar fraction (%) of AMS, Y—molar fraction (%) of support.

**Table 2:** Summary of the molar composition of unsupported and supported MgO sorbents.

Sorbent Precursor	Magnesium (mol, %)	Support (mol, %)	Alkali Metal Salts			
			NaNO_3_		(Na,Li,K)NO_3_	
			(mol, %)	Sample ID	(mol, %)	Sample ID
MgO-SG ^1^	100	0	15	15 Na-MgO	15	15 (Na,K,Li)-MgO
			25	25 Na-MgO	25	25 (Na,K,Li)-MgO
			35	35 Na-MgO	35	35 (Na,K,Li)-MgO
MgO-Ca-SG	90	10			15	15 (Na,K,Li)-MgO-Ca
MgO-Ce-SG	90	10			15	15 (Na,K,Li)-MgO-Ce

^1^ SG: sol gel.

**Table 3:** Average crystallite size of MgO-SG undoped and doped with 15, 25 and 35% (mol) of AMS.

AMS	Average Crystallite Size (nm) of MgO-SG Undoped and Doped with AMS (%, mol)			
	0	15	25	35
NaNO_3_	10	15	15	16
(Na, Li, K) NO_3_		26	28	29

**Table 4:** Average crystallite size (nm) of unsupported and supported MgO-SG undoped and doped with 15% of (Na, K, Li)NO_3_.

Unsupported and Supported MgO-SG Sorbents	Average Crystallite Size (nm) of Sorbents Undoped and Doped with 15% of (Na, K, Li)NO_3_	
	0	15
MgO-SG	10	26
MgO-SG-Ca	12	30
MgO-SG-Ce	12	27

**Table 5:** Thermal decomposition of mono and ternary alkali salts under air and CO_2_ atmosphere: working range and decomposition range (°C).

Sample	Atmosphere	T3 (°C)	Melting Point (°C)	Working Range (°C)	Decomposition Range (°C)
NaNO_3_	Air	620	308 [11]	308–620	620–814
	CO_2_	581		308–581	581–731
(Na, Li, K)NO_3_	Air	575	120 [28]	120–575	575–818
	CO_2_	595		120–595	595–736

**Table 6:** Properties of MgO sorbent undoped and doped with different molar fractions of mono or ternary AMS: maximum instantaneous rate of change in temperature with respect to CO_2_ uptake (°C), inflection temperature (°C), maximum CO_2_ uptake temperature (°C), CO_2_ uptake range (°C) and maximum CO_2_ uptake (mg CO_2_ g^−1^ sorbent).

MgO-Based Sorbent	Undoped	Doped					
		NaNO_3_ (mol, %)		(Na, Li, K)NO_3_ (mol, %)			
		15	25	35	15	25	35
Maximum instantaneous rate of change in temperature with respect to CO_2_ uptake (°C)	-	310	304	310	322	319	Peak not defined
Inflection temperature (°C)	-	234	237	240	175	184	Not detected
Maximum CO_2_ uptake temperature (°C)	-	399	396	395	391	388	388
CO_2_ uptake temperature range (°C)	20–50	234–399	237–396	240–395	175–391	184–388	-
Maximum CO_2_ uptake (mg CO_2_ g^−1^ sorbent)	36	165	231	292	239	161	139

**Table 7:** Properties of unsupported and supported MgO sorbents (Ca or Ce-based support) undoped and doped with 15% of (Na, Li, K)NO_3_: maximum instantaneous rate of change in temperature with respect to CO_2_ uptake (°C), inflection temperature (°C), maximum CO_2_ uptake temperature (°C), CO_2_ uptake range (°C) and maximum CO_2_ uptake (mg CO_2_ g^−1^ sorbent).

MgO-Based Sorbent	MgO-SG *	MgO-Ca-SG		MgO-Ce-SG	
	% (Na, Li, K)NO_3_				
	15	0	15	0	15
Maximum instantaneous rate of change in temperature with respect to CO_2_ uptake (°C)	322	-	308	-	294
Inflection temperature (°C)	175	-	153	-	210
Maximum CO_2_ uptake temperature (°C)	391	25	384	25	393
CO_2_ uptake temperature range (°C)	175–391	-	153–384	-	210–393
Maximum CO_2_ uptake (mg CO_2_ g^−1^ sorbent)	239	73	299	86	252

* Values from Table 6 for comparison.

## Data Availability

All data are reported in the paper.

## Supplementary Materials

The following supporting information can be downloaded at https://www.mdpi.com/article/10.3390/ma16247539/s1, Figure S1: Illustration of 1st and 2nd derivative of MgO sorbent doped with different molar fractions of mono or ternary alkali metal salts; Figure S2: Carbonation profile of MgO-SG sorbent impregnated with 15% of (Na, Li, K)NO_3_; Figure S3: Illustration of 1st and 2nd derivative of supported MgO sorbent (Ca- and Ce- support) doped with different molar fractions of mono or ternary alkali metal salts.
