# Supplementary material for: Understanding the Role of Mono and Ternary Alkali Metal Salts on CO2 Uptake of MgO Sorbents
Source: Materials (Basel). 2023 Dec 6;16(24):7539. doi: 10.3390/ma16247539 (PMC10744914; doi:10.3390/ma16247539)
Supplement: Supplementary file 1 [file materials-16-07539-s001.zip › materials-2656831-supplementary.pdf]

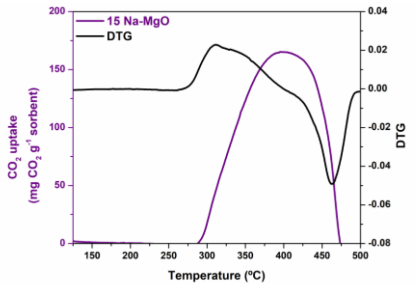


**(A).** $CO_2$ uptake profile of MgO-SG sorbent doped with 15 % of $NaNO_3$ and respective first derivative.

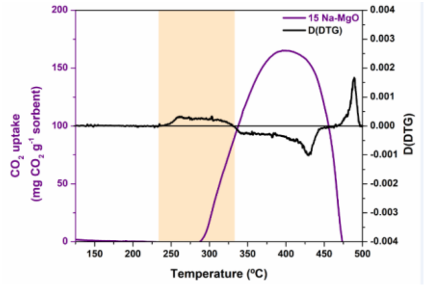


**(B).** $CO_2$ uptake profile of MgO-SG sorbent doped with 15 % of $NaNO_3$ and respective second derivative (acelerating sorption is highlight in yellow).

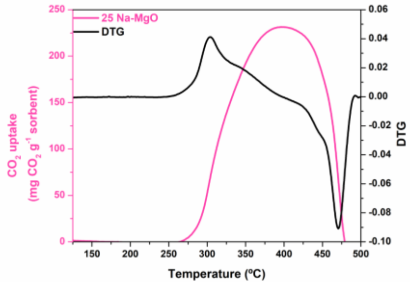


**(C).** $CO_2$ uptake profile of MgO-SG sorbent doped with 25 % of $NaNO_3$ and respective first derivative.

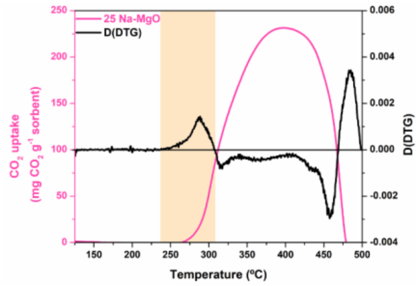


**(D).** $CO_2$ uptake profile of MgO-SG sorbent doped with 25 % of $NaNO_3$ and respective second derivative (acelerating sorption is highlight in yellow).

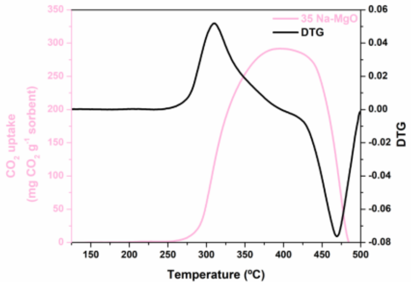


**(E).** $CO_2$ uptake profile of MgO-SG sorbent doped with 35 % of $NaNO_3$ and respective first derivative.

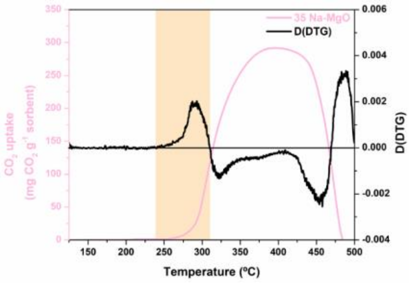


**(F).** $CO_2$ uptake profile of MgO-SG sorbent doped with 35 % of $NaNO_3$ and respective second derivative (acelerating sorption is highlight in yellow).

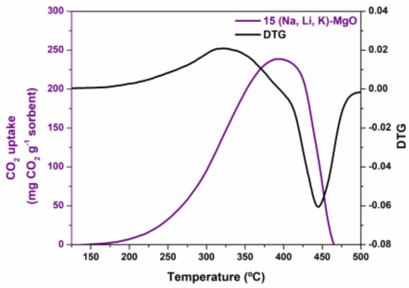


**(G).** $CO_2$ uptake profile of MgO-SG sorbent doped with 15 % of (Na, K, Li)$NO_3$ and respective first derivative.

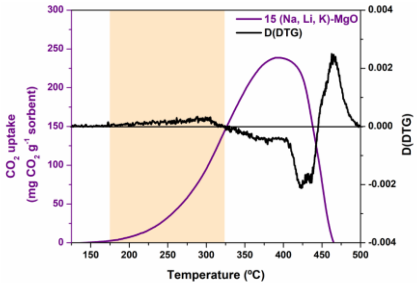


**(H).** $CO_2$ uptake profile of MgO-SG sorbent doped with 15 % of (Na, K, Li)$NO_3$ and respective second derivative (acelerating sorption is highlight in yellow).

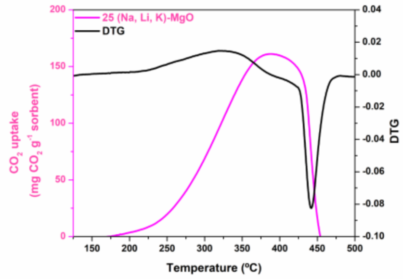


**(I).** $CO_2$ uptake profile of MgO-SG sorbent doped with 25 % of (Na, K, Li)$NO_3$ and respective first derivative.

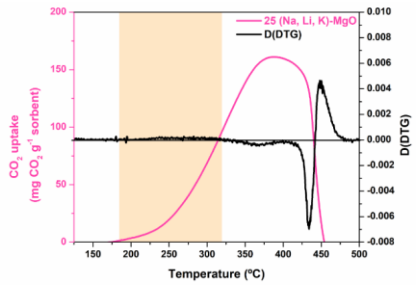


**(J).** $CO_2$ uptake profile of MgO-SG sorbent doped with 25 % of (Na, K, Li)$NO_3$ and respective second derivative (acelerating sorption is highlight in yellow).

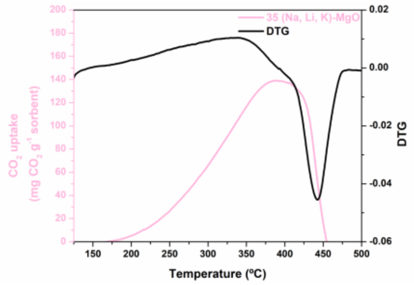


**(K).** $CO_2$ uptake profile of MgO-SG sorbent doped with 35 % of (Na, K, Li)$NO_3$ and respective first derivative.

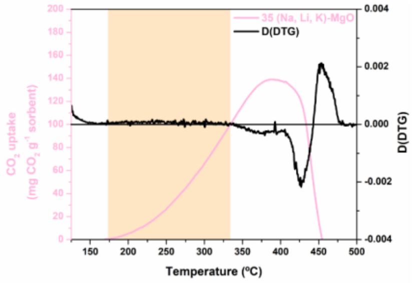


**(L).** $CO_2$ uptake profile of MgO-SG sorbent doped with 35 % of (Na, K, Li)$NO_3$ and respective second derivative (acelerating sorption is highlight in yellow).

**Figure S1.** Illustration of 1st and 2nd derivative of MgO sorbent doped with different molar fractions of mono or ternary alkali metal salts. These data allow to determine the sorbents maximum instantaneous rate of change of temperature with respect to $CO_2$ uptake (°C), inflection temperature (°C), maximum $CO_2$ uptake temperature (°C), $CO_2$ uptake range (°C) and maximum $CO_2$ uptake (mg $CO_2$ $g^{-1}$ sorbent).

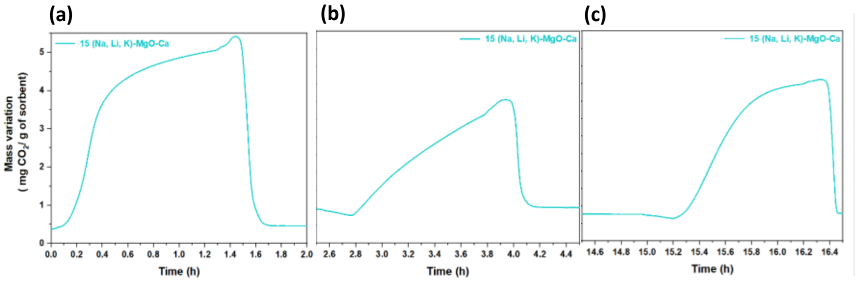


**Figure S2.** Carbonation profile of MgO-SG sorbent impregnated with 15% of (Na, Li, K)$NO_3$. Profile of carbonation (300 °C, 60 min) of MgO-SG sorbent impregnated with 15% of (Na, Li, K)$NO_3$: (a) 1st cycle, (b) 2nd cycle, (c) 7th cycle

**Commented [M1]:** This is the original picture. Please confirm whether the current position is reversed. If so, please modify it.

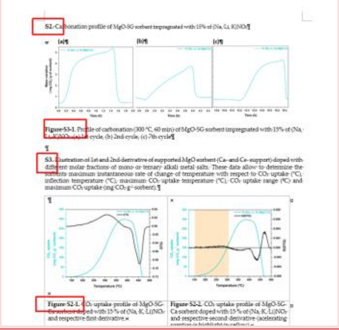

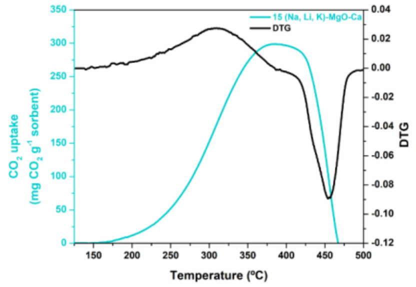


**(A).** $CO_2$ uptake profile of MgO-SG-Ca sorbent doped with 15 % of (Na, K, Li)$NO_3$ and respective first derivative.

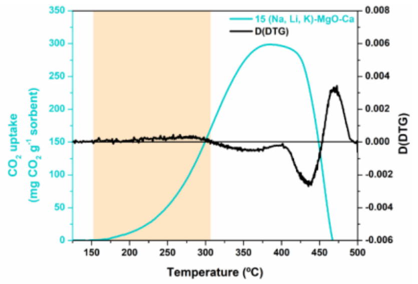


**(B).** $CO_2$ uptake profile of MgO-SG-Ca sorbent doped with 15 % of (Na, K, Li)$NO_3$ and respective second derivative (acelerating sorption is highlight in yellow).

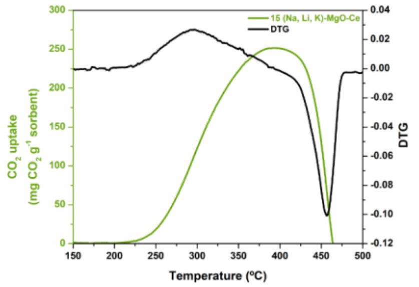


**(C).** $CO_2$ uptake profile of MgO-SG-Ce sorbent doped with 15 % of (Na, K, Li)$NO_3$ and respective first derivative.

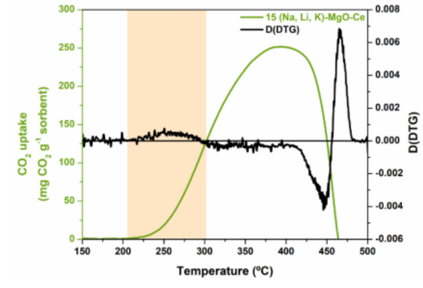


**(D).** $CO_2$ uptake profile of MgO-SG-Ce sorbent doped with 15 % of (Na, K, Li)$NO_3$

and respective second derivative (acelerating sorption is highlight in yellow).

**Figure S3.** Illustration of 1st and 2nd derivative of supported MgO sorbent (Ca- and Ce- support) doped with different molar fractions of mono or ternary alkali metal salts. These data allow to determine the sorbents maximum instantaneous rate of change of temperature with respect to $CO_2$ uptake (°C), inflection temperature (°C), maximum $CO_2$ uptake temperature (°C), $CO_2$ uptake range (ºC) and maximum $CO_2$ uptake (mg $CO_2$ $g^{-1}$ sorbent).
